# Identifying transferable lessons from cholera epidemic responses by Médecins Sans Frontières in Mozambique, Malawi and the Democratic Republic of Congo, 2015–2018: a scoping review

**DOI:** 10.1186/s13031-022-00445-1

**Published:** 2022-03-29

**Authors:** Lauren D’Mello-Guyett, Oliver Cumming, Elliot Rogers, Rob D’hondt, Estifanos Mengitsu, Maria Mashako, Rafael Van den Bergh, Placide Okitayemba Welo, Peter Maes, Francesco Checchi

**Affiliations:** 1 Department of Disease Control, Faculty of Infectious and Tropical Diseases, London School of Hygiene and Tropical Medicine, London, UK; 2 Médecins Sans Frontières, Kinshasa, Democratic Republic of Congo; 3 Environmental Health Unit, Médecins Sans Frontières, Brussels, Belgium; 4 LuxOR, Luxembourg Operational Research Unit, Médecins Sans Frontières, Luxembourg, Luxembourg; 5 Programme National d’Elimination du Choléra et de lutte contre les autres Maladies Diarrhéiques, Kinshasa, Democratic Republic of Congo; 6 WASH Section, UNICEF, Kinshasa, Democratic Republic of Congo; 7 Department of Infectious Disease Epidemiology, Faculty of Epidemiology and Population Health, London School of Hygiene and Tropical Medicine, London, UK

**Keywords:** Cholera, Outbreaks, Emergency, Water, Sanitation, Hygiene, Oral cholera vaccination

## Abstract

**Background:**

Cholera epidemics occur frequently in low-income countries affected by concurrent humanitarian crises. Evaluations of these epidemic response remains largely unpublished and there is a need to generate evidence on response efforts to inform future programmes. This review of MSF cholera epidemic responses aimed to describe the main characteristics of the cholera epidemics and related responses in these three countries, to identify challenges to different intervention strategies based on available data; and to make recommendations for epidemic prevention and control practice and policy.

**Methods:**

Case studies from the Democratic Republic of Congo, Malawi and Mozambique were purposively selected by MSF for this review due to the documented burden of cholera in each country, frequency of cholera outbreaks, and risk of humanitarian crises. Data were extracted on the characteristics of the epidemics; time between alert and response; and, the delivery of health and water, sanitation and hygiene interventions. A Theory of Change for cholera response programmes was built to assess factors that affected implementation of the responses.

**Results and conclusions:**

20 epidemic response reports were identified, 15 in DRC, one in Malawi and four in Mozambique. All contexts experienced concurrent humanitarian crises, either armed conflict or natural disasters. Across the settings, median time between the date of alert and date of the start of the response by MSF was 23 days (IQR 14–41). Almost all responses targeted interventions community-wide, and all responses implemented in-patient treatment of suspected cholera cases in either established health care facilities (HCFs) or temporary cholera treatment units (CTUs). In three responses, interventions were delivered as case-area targeted interventions (CATI) and four responses targeted households of admitted suspected cholera cases. CATI or delivery of interventions to households of admitted suspected cases occurred from 2017 onwards only. Overall, 74 factors affecting implementation were identified including delayed supplies of materials, insufficient quantities of materials and limited or lack of coordination with local government or other agencies. Based on this review, the following recommendations are made to improve cholera prevention and control efforts: explore improved models for epidemic preparedness, including rapid mobilisation of supplies and deployment of trained staff; invest in and strengthen partnerships with national and local government and other agencies; and to standardise reporting templates that allow for rigorous and structured evaluations within and across countries to provide consistent and accessible data.

**Supplementary Information:**

The online version contains supplementary material available at 10.1186/s13031-022-00445-1.

## Introduction

Across Sub-Saharan Africa, an estimated 430 million people are at risk of cholera [1]. Annually, there are an estimated 1.3–4.0 million cases of cholera worldwide resulting in between 21,000 and 143,000 deaths [1]. Many of the largest epidemics on the continent have occurred concurrently with armed conflict and ongoing humanitarian crises [2–5]. Cholera epidemics can evolve rapidly and most often occur in settings with limited surveillance systems to detect the outbreak’s onset.

By 2030, the Global Task Force on Cholera Control (GTFCC) has set a target to reduce cholera mortality by 90% and eliminate cholera in 20 out of 47 countries by 2030 [6]. The renewed focus on cholera provides a framework for synchronising the efforts of countries, donors, implementing agencies and support coordinated multisectoral implementation of cholera control measures [7]. The strategy has three axes: (1) to focus on cholera hotspots in endemic countries with well targeted interventions; (2) to reinforce early detection and response to contain epidemics quickly; and (3) to provide an effective mechanism for coordinated technical support, financing and resources at the global and country level [8]. There are five pillars of the GTFCC including surveillance, case management, oral cholera vaccination (OCV), water, sanitation and hygiene (WASH), and community engagement. All pillars interact and play an integral role in multisector responses in short-term, emergency responses and for longer term sustainable elimination of the disease.

Progress towards this target set by the GTCC may benefit from critical review of past responses, particularly as low- and middle-income country (LMIC) governments, including Democratic Republic of Congo (DRC), Malawi and Mozambique, develop multisectoral National Cholera Plans (NCPs) to address cholera within their contexts [9]. Typically, interventions to prevent and control cholera epidemics have varied between mass, community-wide campaigns, in which multisector interventions are aligned to other WASH-related disease control efforts and aim to prevent the recurrence of outbreaks [10], to household-level or case-area targeted interventions (CATIs), in which services are delivered to a defined area surrounding a case to take advantage of the natural clustering of cases within a given radius, so as to contain or extinguish the outbreaks [11–15]. Whilst cholera responses will always be specific to the geographical and social context, it is important that the operational constraints for delivering timely interventions are documented and evaluated. Previous reviews have shown a dearth of evaluations of epidemic responses, depriving governmental and other response actors of an evidence base for improving practice and a baseline against which to track progress [16, 17]. In particular, delayed detection, confirmation and response can considerably dampen the impact of CATI-like approaches, with delays of > 2 weeks expected to result in spill-over beyond the initial outbreak cluster [18]; interventions that seek to contain outbreaks before they propagate widely, including case-based or localised distribution of hygiene kits [19] and vaccination [20, 21] are particularly dependent on early response, as is case management of cholera cases [22, 23].

Over the last five decades, the international non-governmental organisation (NGO), Médecins Sans Frontières (MSF) has intervened in multiple cholera epidemics in crisis-affected Sub-Saharan African settings. In this review, we present three countries (DRC; Malawi; and Mozambique) as case studies of MSF’s response to cholera epidemics during the period 2015–2018. The aim of this study was to describe the main characteristics of the cholera epidemics and related responses in these three countries, to identify challenges of different intervention strategies based on available data; and to make recommendations for epidemic prevention and control practice and policy.

## Methods

### Study design, inclusion and exclusion criteria

This review analysed cholera response reports by MSF. Intervention reports were eligible for inclusion if they were finalised during 2015–2018 and described or evaluated the organisation’s intervention during a cholera outbreak. Mozambique, Malawi and DRC were purposively selected due to the documented burden of cholera in each of the countries [6], frequency of cholera outbreaks [5]; and risk of humanitarian crises [3, 4, 24]. World Health Organization (WHO) definitions for a cholera alert were used in both the responses and this analysis [25]. The review is reported according to the Preferred Reporting Items for Systematic Reviews and Meta‐Analyses extension for Scoping Reviews (PRISMA-ScR) guidelines [26] (Additional file 1). The review was not pre-registered prior to publication.

### Theory of change

**Fig. 1:**
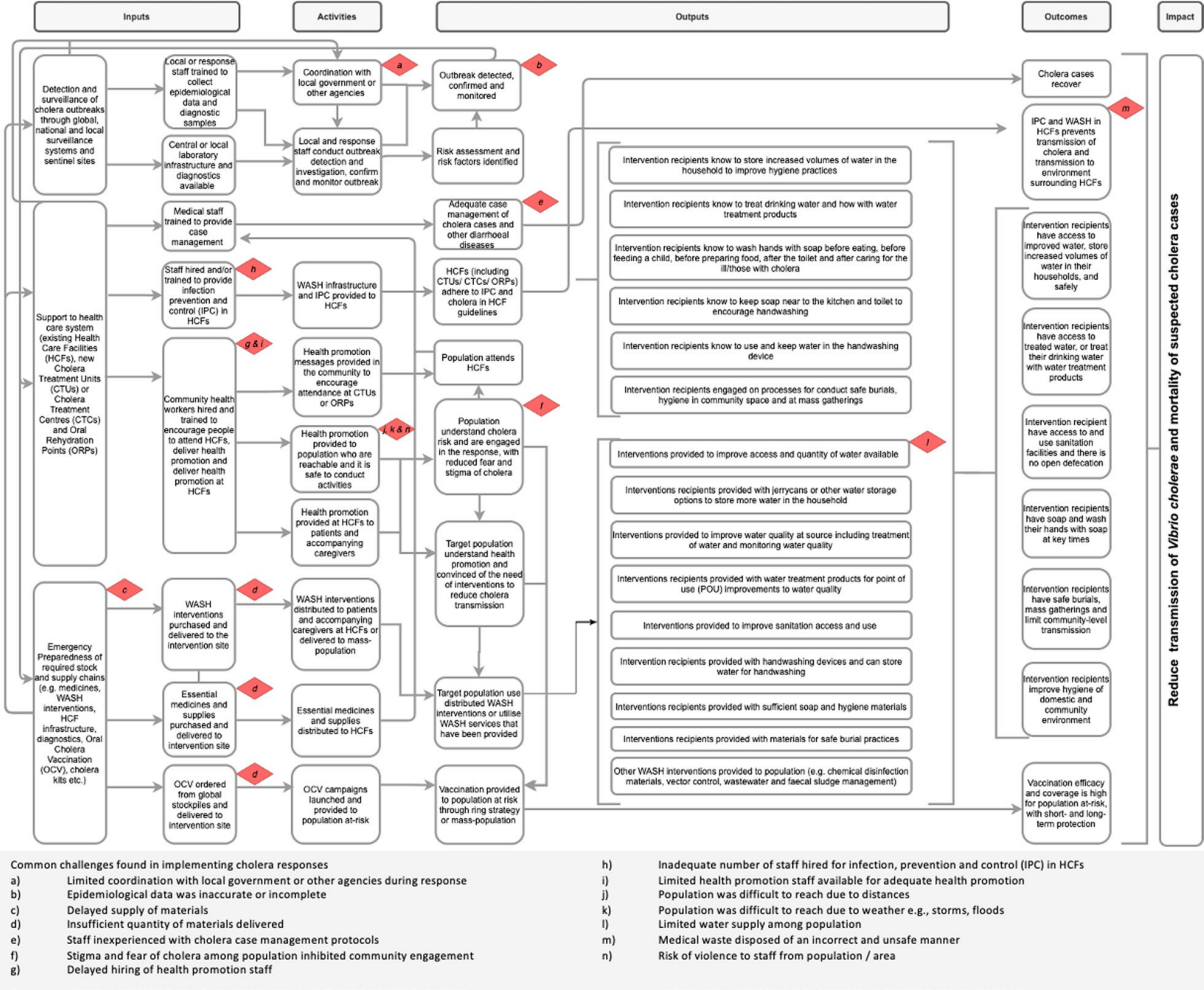
Theory of Change of cholera response programmes, with challenges identified in the case studies in DRC, Mozambique and Malawi, 2015–2018

To guide the review, we developed a Theory of Change (ToC) diagram to identify requirements for implementing a cholera epidemic response, and pathways whereby the intended effects on cholera transmission and disease burden (Impact) may be achieved and/or influenced by common challenges (Fig. 1). ToC models can be useful to create alongside evaluations of programmes as they can provide insights into the key bottlenecks and constraints of programme implementation [27–29]. This ToC was developed with inputs from the study team and acts as a framework by which we can understand the barriers within the past responses. The ToC Inputs are (1) national and local emergency preparedness supplies, the supply of interventions to the intervention site and national and local surveillance systems that can detect the outbreak; these determine (2) adequate health promotion and timely provision of the interventions to the target population (Activities); which in turn lead to (3) the target population understanding the health promotion and intending to accept or utilise the interventions (Outputs); (4) intervention recipients that are motivated and have the ability to practice the target behaviour/s or access cholera control services (Outputs); and, finally to (5) a reduction in transmission of *Vibrio cholerae* and mortality of cholera cases (Outcomes and Impact).

### Data extraction and analysis

Documents were shared by MSF’s operational desks overseeing programmes in the three countries, and were transferred to Endnote X9 (Clarivate Analytics, Boston, USA). Reports were screened according to the inclusion criteria described and data extracted by two reviewers into an MS Excel (Microsoft, Redmond, VA, USA) sheet. Data extraction was reviewed by two co-authors (LDG and ER) and any disagreements agreed by consensus.

Qualitative operational factors that affected the response were extracted from reports and analysis was conducted in MS Excel (Microsoft, Redmond, VA, USA). Based on thematic content analysis [30, 31], common challenges were identified and coded deductively according to pathways to the ToC. These have been mapped to Fig. 1 to show the key challenges affecting an effective cholera response programme.

Data were extracted on the following, as available:

1. Description of the outbreak (e.g., country, geographical setting, transmission, and number of cases and deaths);
2. Time lag from alert (i.e., through formal or informal surveillance systems) or onset (i.e., report of a suspected cholera case meeting the WHO definition) to response (i.e., initiation of health and/or WASH interventions for prevention and control of cholera, as specified in Table 1);
3. Specific health and WASH interventions implemented (Table 1);
4. Population targeted by interventions (e.g., community-wide interventions, CATI, households of cases or only cases treated at health care facilities, HCF);
5. Operational factors mentioned in the report that affected the response, organised thematically based on a Theory of Change (ToC) of cholera responses (see below and Fig. 1).

**Table 1:** Categories and definitions of cholera prevention and control interventions included in the review

Health interventions [32]	Case management	Treatment is based on the degree of dehydration of the patient: no dehydration, some dehydration or severe dehydration. Patients with no signs or some signs of dehydration are treated with ORS (plan A and plan B, respectively). Patients with severe dehydration require IV rehydration (Plan C). Antibiotics are indicated in patients with severe dehydration and, in patients with high purging or treatment failure or in patients with coexisting conditions or comorbidities. In children aged 6 months to 5 years, zinc supplementation (20 mg p.o. zinc sulphate per day for 10 days) should be started immediately [32]
	Oral Cholera Vaccination (OCV)	Any of the two types of OCV, WC-rBS, killed whole cell monovalent (O1) vaccines with a recombinant B subunit of cholera toxin (Dukoral®) and (ii) WC, killed modified whole cell bivalent (O1 and O139) vaccines without the B subunit (Shanchol™, Euvichol® and mORCVAX™), currently available and recommended by WHO [33]. Vaccination campaigns are guided by a series of criteria, governed by the GTFCC [20]
	Antibiotic chemoprophylaxis	Any antibiotic chemoprophylaxis, with doxycycline, azithromycin or ciprofloxacin, is currently not recommended by WHO. However, selective prophylaxis of household contacts of cholera cases (i.e., considered at high risk of being infected with *Vibrio cholerae*) has been implemented in the past
	Other health interventions	As applicable
WASH interventions [10]	Improving the access to water sources and/or quantity of water	Any intervention to provide a new and/or improved water supply or distribution system, or both, i.e., to reduce direct and indirect exposure with contaminated water (e.g., installation of piped water supply, hand pumps, boreholes; installation or extension of distribution networks; water trucking or tankers; and protection of water sources)
	Improving the quality of water: water treatment at source	Any intervention to improve the microbiological quality of drinking water at the source, including: assessment and monitoring of water quality i.e., microbiological, chemical and physical quality removing or inactivating microbiological pathogens (e.g., water source level water treatment systems, filtration, sedimentation, chemical treatment, heat treatment, ultraviolet (UV) radiation or flocculation)
	Improving the quality of water: point of use (POU) and safe storage	Any intervention to expand use of or improve the microbiological quality of drinking water at the point of use (POU), including: Assessment and monitoring of water quality i.e., microbiological, chemical and physical quality Protecting the microbiological quality of water prior to consumption (e.g., chemical treatment, filtration, heat treatment, flocculation, UV radiation, residual disinfection, protected distribution, improved storage)
	Improving the access to and use of sanitation facilities and reducing exposure to faeces	Any intervention to introduce, improve or expand the coverage of facilities for the safe management, disposal and treatment of excreta, i.e., to reduce direct and indirect contact with human faeces, and to promote the use of sanitation facilities by the population (e.g., latrine construction, pour flush, composting or water sealed flush toilet, piped sewer system, septic tank, simple pit latrines, VIP latrine, defecation trenches or use of a potty or scoop for the disposal of child faeces)
	Behaviour change interventions to improve personal, domestic and food hygiene practices	Any intervention to improve hygiene, including: Promotion of hygiene behaviours, norms or practices surrounding personal, food and hand hygiene Assessment and monitoring of hygiene behaviours, norms or practices, including adaptation of activities Any named method of delivery of hygiene promotion (e.g., interpersonal channels, house-to-house visits, community meetings, mass and social media, targeted areas or information, education and communication (IEC) materials, or other hygiene promotion activities) Any named theory, framework or technique for hygiene promotion (e.g., behaviour change communication (BCC), community engagement, social marketing and demand creation, integrated hardware)
	Distribution of hygiene materials or non-food items (NFIs)	Any intervention that provides hygiene materials or use of hygiene materials (e.g., soap, hygiene kits, handwashing stands, sinks and other facilities)
	Promotion or distribution of disinfection and cleaning of households and community spaces and/or materials	Any intervention that provides or distributes disinfection materials (e.g., chlorine spraying, disinfection of clothes, disinfectants, disinfection of bedding or vehicles) or promotes household cleaning (e.g., safe laundry practices, cleaning of floors and furniture)
	Improving dead body management and safe funeral practices	Any intervention to improve safe funeral practices, funeral gatherings and management of corpses in the community
	Improving the management of wastewater and faecal sludge	Any intervention to improve management of wastewater and faecal sludge
	Provision of interventions that improve solid waste disposal	Any intervention to improve solid waste disposal, particularly in public places
	Use of vector control interventions to reduce flies	Any intervention to improve fly control and/or other vectors
	Other WASH interventions	As applicable

## Results

### Description of outbreaks and intervention sites

Twenty outbreak response reports, of the 35 provided by MSF, met the inclusion criteria. Of the 15 reports that were excluded from the analysis, eight were incomplete and did not provide enough data for extraction and seven were duplicative reports from the same outbreak but written by another person. There were 15 included reports from DRC, four from Mozambique and one from Malawi. Twelve outbreaks were in rural settings, seven in urban and one in both. All contexts experienced concurrent humanitarian crises, either armed conflict or natural disasters. In Mozambique, all outbreaks were among populations affected by armed conflict with one outbreak among a population affected by armed conflict and a natural disaster (flooding and cyclones). In Malawi, the outbreak started among displaced populations, and, in DRC, all outbreaks were in areas with ongoing medium-intensity conflict. All 20 reports reported the date of the initial outbreak alert, but no report detailed the date of onset of symptoms in the first identified cases. Alerts came through both formal surveillance systems (70%, n = 14) and informal sources (30%, n = 6), and all were additionally investigated by MSF. Formal surveillance systems included routine health surveillance systems and sentinel sites. Informal sources included community-based reporting from community health workers (CHWs) to a local HCF, typically based on report of a person with suspected symptoms of cholera. Two of the alerts were confirmed by culture (10%, n = 2), half were reported as confirmed but the authors did not specify if this was by culture (50%, n = 10) and in two instances the alerts reported the use of RDTs, but it was unclear if this was for confirmation of the alert or for general monitoring of the outbreak (10%, n = 2). Other characteristics of the outbreaks such as previous MSF operations, locations and/or distances to HCFs, or length of the outbreaks were not included in any of the reports. Characteristics of the outbreaks including cumulative cases, cumulative deaths and case fatality rate (CFR) are reported in Table 2.

**Table 2:** Description of outbreaks in the Democratic Republic of Congo, Malawi and Mozambique between 2015 and 2018

Country	Year	District	Geographical context	Transmission	Political instability^a^	Cumulative ascertained cases	Cumulative ascertained deaths	Case fatality ratio (%)	Alert confirmed by
DRC	2015	Maniema	Urban	Endemic	Medium-intensity conflict	3316	70	2.1	Confirmed but not specified
DRC	2016	Maniema	Rural	Endemic	Medium-intensity conflict	319	16	5.0	Confirmed but not specified
DRC	2016	Tshopo	Rural	Endemic	Medium-intensity conflict	1 758	241	13.7	Confirmed but not specified
DRC	2016	Tshopo	Rural	Endemic	Medium-intensity conflict	72	3	4.2	Culture
DRC	2016	Tshopo	Rural	Endemic	Medium-intensity conflict	688	44	6.4	Confirmed but not specified
DRC	2016	Tshopo	Rural	Endemic	Medium-intensity conflict	137	11	8.0	Confirmed but not specified
DRC	2016	Mongala	Rural	Endemic	Medium-intensity conflict	12 292	32	0.3	Culture
DRC	2016	Equateur	Rural	Endemic	Medium-intensity conflict	620	20	3.2	Confirmed but not specified
DRC	2016	Kinshasa	Urban	Endemic	Medium-intensity conflict	8	1	12.5	Confirmed but not specified
DRC	2016	Kinshasa	Urban	Endemic	Medium-intensity conflict	15	3	20.0	Confirmed but not specified
DRC	2017	Kongo Lomami	Rural	Endemic	Medium-intensity conflict	786	60	7.6	Not specified
DRC	2018	Kasaï-Oriental	Urban	Endemic	Medium-intensity conflict	130	0	0.0	Not specified
DRC	2018	Kasaï-Oriental	Rural	Endemic	Medium-intensity conflict	666	32	4.8	Confirmed but not specified
DRC	2018	Kinshasa	Urban	Endemic	Medium-intensity conflict	1 153	35	3.0	Not specified
DRC	2018	Mai Ndombe	Urban/Rural	Endemic	Medium-intensity conflict	473	28	5.9	Not specified
Malawi	2015	Nhamayabue and Caia	Rural	Endemic	Population displacement	489	2	0.4	Confirmed but by use of RDT
Mozambique	2015	Mocuba	Urban	Endemic	Armed conflict	317	2	0.6	Not specified
Mozambique	2015	Tete	Urban	Endemic	Armed conflict	3591	22	0.6	Not specified
Mozambique	2017	Meconta and Monapo	Rural	Endemic	Armed conflict / Natural disaster	607	0	0.0	Confirmed but not specified
Mozambique	2018	Memba	Rural	Endemic	Armed conflict	409	2	0.5	Confirmed but by use of RDT

^a^Political instability defined by World Bank Fragile and conflict-affected situations list FY06 to FY20 [3], Complex Emergency Database [24] and International Disaster Database [4]

### Time from alert to response

Across the three countries, median time from the date of the alert to date of case confirmation was 10 days (IQR 3–28). The median time between the date of alert and date of the start of the response by MSF was 23 days (IQR 14–41). Among the eight reports that also reported when WASH interventions started, there was a further median delay of 8 days (IQR 6–12) from the launch of a response to the launch of any WASH intervention. Median time from alert to response overall and by country are reported in Table 3.

**Table 3:** Median delays (with interquartile range (IQR) and range) between cholera alerts, confirmation, response and launch of water, sanitation and hygiene (WASH) interventions in the Democratic Republic of Congo, Malawi and Mozambique, 2015–2018

Country	Year range	Median delay between alert and confirmation	IQR (days)	Range (days)	Median delay between alert and response	IQR (days)	Range (days)	Median delay between response and launch of any WASH intervention	IQR (days)	Range (days)
All	2015–2018	10	3–28	1–76	23	14–41	2–126	8	6–12	0–14
DRC	2015–2018	7	3–25	1–76	22	16–42	4–78	7	5–7	3–8
Malawi	2015	42	42	42	10	6–21	2–31	12	12	12
Mozambique	2015–2018	12	6–18	0–24	37	27–64	15–126	13	9–14	5–14

### Delivery of health and WASH interventions

Almost all responses targeted interventions community-wide, and all responses implemented in-patient treatment of suspected cholera cases in either established HCFs or temporary cholera treatment units (CTUs). In three responses, interventions were delivered as CATI and four responses targeted households of admitted suspected cholera cases. CATI responses or delivery of interventions to households of admitted suspected cases occurred from 2017 onwards only (Table 4).

**Table 4:**
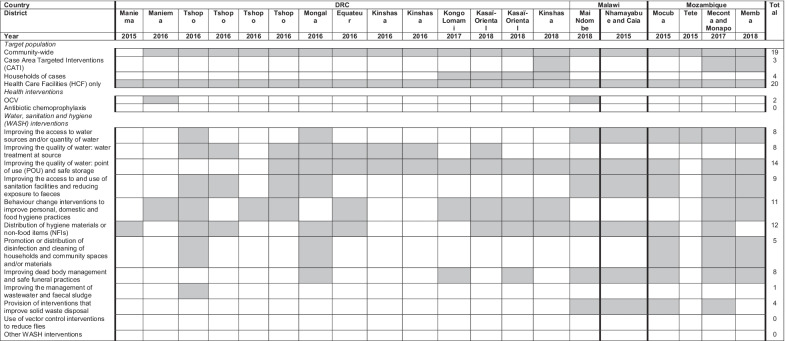
Implementation of cholera responses in the Democratic Republic of Congo, Malawi and Mozambique, 2015–2018

*HCF* Health Care Facilities, *CATI* Case Area Targeted Interventions, *OCV* Oral Cholera Vaccination, *POU* Point of Use, *WASH* Water, Sanitation and Hygiene

Among the 20 responses, OCV was deployed only twice (both in DRC) and no response across the three countries included antibiotic chemoprophylaxis. WASH interventions deployed across responses varied between years and contexts, but the most widely implemented interventions were distribution of point-of-use (POU) water treatment, distribution of non-food items (NFIs) and behaviour change interventions that promoted hand washing with soap (Table 4).

### Common challenges affecting cholera responses

For each outbreak, challenges that delayed the response and affected the implementation of programme activities were extracted from reports and mapped to the ToC (Fig. 1). Overall, 74 challenges were identified across reports: 33 from DRC, 29 from Mozambique and 12 from Malawi (Table 5). Among the Inputs for programme delivery, delayed supplies of materials (c) and hiring or availability of health promotion staff (g and i) were frequently cited as challenges. In addition to delays, insufficient quantities of materials were delivered for effective programme delivery (d) and an insufficient number of key staff hired, particularly staff for infection, prevention and control (IPC) in the HCFs (h). The most widely reported challenge affecting Activities was the limited or lack of coordination with local government or other agencies (a), which is likely related to the second most commonly mentioned challenge of incomplete or inaccurate epidemiological data collection during the response (b), which could have affected timeliness of response initiation, the appropriateness of the response strategy and real-time adaptation of the response. Activities were also affected by the distances between households and HCFs and the ability to reach the population during bad weather or insecurity (j, k and n). Among Outputs, adequate case management was constrained by staff inexperience with cholera case management protocols (e) and in some cases, the response was affected by stigma and fear of cholera among the population (f). Other aspects of programme delivery that were frequently reported as challenging included limited water supply available to the population (l), which may affect their ability to adopt hygiene behaviours, and the inadequate medical waste management in HCFs, which could pose a nosocomial transmission risk (m).

**Table 5:** Common challenges found in implementing cholera responses, by country

	DRC	Mozambique	Malawi	Total
Number of reports (n)	15	4	1	20
Coordination with government or other agencies				
(a) Limited coordination with local government or other agencies during response	7	6	1	14
Surveillance				
(b) Epidemiological data was inaccurate or incomplete	6	3	1	10
Supply				
(c) Delayed supply of materials	5	1	1	7
(d) Insufficient quantity of materials delivered	6	1	2	9
Case management				
(e) Staff inexperienced with cholera case management protocols	3	1	2	6
Community engagement				
(f) Stigma and fear of cholera among population inhibited community engagement	–	4	1	5
Human resources				
(g) Delayed hiring of health promotion staff	2	2	–	4
(h) Inadequate number of staff hired for infection, prevention and control (IPC) in HCFs	–	1	2	3
(i) Limited health promotion staff available for adequate health promotion	1	2	–	3
Geographical context				
(j) Population was difficult to reach due to distances	1	2	–	3
(k) Population was difficult to reach due to weather e.g., storms, floods	–	1	–	1
Water supply				
(l) Limited water supply among population	1	1	1	3
Medical waste management at health care facilities (HCFs)				
(m) Medical waste disposed of an incorrect and unsafe manner	–	2	1	3
Safety and security				
(n) Risk of violence to staff from population/area	1	2	–	3
Total	33	29	12	74

## Discussion

Responses to cholera epidemics among populations affected by humanitarian crises can be complex, comprising multiple interacting components, and are often applied in both urban and rural contexts. The case studies included in this review relate to largescale responses by one major humanitarian agency to cholera epidemics in three countries. The package of interventions mobilised for each response varied but common challenges were identified. The findings from this review are discussed in relation to three key areas: (1) characteristics of the included cholera epidemics; (2) time from alerts to response experienced by MSF; and (3) delivery of interventions and factors affecting implementation.

### Characteristics of included cholera epidemics

All epidemics reviewed here occurred in identified cholera hotpots in Sub-Saharan Africa [21], which have documented weak surveillance systems [34], low coverage of WASH services [35] and high global acute malnutrition (GAM) prevalence (2.7%, 6.1%, and 8.10% in Malawi, Mozambique and DRC, respectively [36]), which all increase risk and severity of outbreaks. Additionally, presence of armed conflict and population displacement likely exacerbated *V. cholerae* transmissibility and case fatality among symptomatic cases, as demonstrated by other research documenting the overlap between crises and cholera epidemics [37–40]. CFR was high in DRC compared to Mozambique and Malawi; however, any comparison may be erroneous as it does not reflect data from individual treatment centres, and varying levels of misclassification and under-ascertainment may apply to both the numerators and denominators to calculate CFRs. Additional efforts to estimate overall CFR need to be undertaken during and after outbreaks to identify the circumstances where cholera cases are dying and to develop strategies to prevent those deaths.

### Time from alert to response

Overall, from 2015 to 2018, median time from epidemic alert to confirmation, by national surveillance systems, was 10 days. Between alert and response by MSF, median time was 22.5 days. In Malawi, DRC and Mozambique, up to 31, 78 and 126 days were observed from alert to response by MSF, respectively. Epidemic detection and time to response in our review of MSF data were comparatively longer compared to a recently published review by Ratnayake et al. [18], which found delays of 5 days between alert and confirmation and 10 days between alert and response, but similar to a review by Bruckner and Checchi [41] of other infectious disease outbreaks in fragile states which showed median delays of 29 days between alert and confirmation or 55 days from alert to response. In most case studies described here, epidemics were confirmed but the methods were not specified. Additionally, our data indicated that epidemiological data were often inaccurate or incomplete: reliance on under-resourced surveillance systems may have led to the data delays in the alert, delayed confirmation and delays to the resulting response [34].

### Delivery of interventions and factors affecting implementation

Delivery of interventions to case-households and/or through CATI was only identified in seven case studies from MSF, all from 2017 onwards. The majority of responses implemented interventions community-wide. Although community-wide interventions are a theoretically equitable approach to intervention delivery, cholera control in outbreaks needs to focus on the dominant transmission pathways between cases and non-cases [10]. Cholera clusters in both space and time within case-households and the areas surrounding case households (~ 150–200 m radius around case households [42]) due to short incubation periods [43], bacterial shedding from both symptomatic and asymptomatic cases [44] and shared WASH risk factors and behaviours [45].

OCV has been recommended since 2010 for epidemic response and in humanitarian crises [46] since 2013, and made available for deployment from a global OCV stockpile, funded by GAVI (the Vaccine Alliance) since 2016. In a recent summary of the 83 deployments and 104 OCV campaigns globally between 2013 and 2018, there were 14 instances where Malawi deployed OCV to the population and 1–3 in DRC [47]. However, only two responses included in this review included vaccination. In both instances in DRC, OCV was provided to MSF through the national government to use leftover and expiring supplies and not as the main intervention strategy. Reports provided little to no information on what factors affected delivery of vaccination in the two instances it was available in DRC. Although OCV is available for deployment, our review highlights that there may still be barriers to humanitarian agencies such as MSF accessing or using those stockpiles and further work is required to understand these challenges. Antibiotic chemoprophylaxis was also not employed in any of the responses, despite being a potentially cost-effective option for cholera control [48].

Among the WASH interventions deployed across the 20 cholera epidemics, the most commonly implemented interventions were the distribution of POU water treatment products, NFIs and behaviour change interventions to promote handwashing with soap. There is considerable evidence to support the effectiveness of these interventions especially when delivered to case-households or areas surrounding suspected cholera cases [49–51], though high coverage needs to be accompanied by efforts to promote and facilitate their use [19]. Generally, the heterogeneity in interventions and the need to come up with a context appropriate response package suggests problems with structured decision-making on the most locally appropriate intervention package. Although guidance for outbreak response has been provided by many agencies [25, 32, 52–57], there is currently no standard package of WASH interventions for cholera outbreaks in humanitarian contexts and there is disagreement between guidelines on intervention strategies [10].

By the time interventions were implemented, it is likely that their potential effects were reduced as some of the reports stated themselves. Limited coordination is not new in outbreak response nor public health, but the frequency in which this challenge was cited across reports indicates that more attention to partnership with local governments and other actors is required both to enhance the ability to detect outbreaks but also to maximise efficiency [6]. Similarly, epidemic preparedness, whereby supplies are pre-positioned or ready to distribute, has been noted as a challenge previously and remains a consistent barrier to feasible and effective cholera control [10]. Supply chain challenges will diminish the effectiveness of any intervention, underscoring the importance of strengthened national and local supply chains [14, 19, 21]. Both supply of materials and human resources were challenges across these case studies and the development of operational models for scaling up both staffing capacity and the supply of materials is an area that could be explored further.

Of immediate concern across the case studies was the reported inexperience of staff with cholera case management protocols and/or lack of training they received if they had little to no experience in cholera case management. Inadequate care was potentially provided to admitted cases, and this will not only affect the CFR among the population [44] but will likely affect the population’s perception and uptake of the intervention [58, 59]. Treatment of cholera cases relies on a strong supply chain of essential medicines and both training and supervision of health care providers on site. Additionally, medical waste management in HCFs needs to be monitored due to both the occupational hazard it poses to both patients and staff and also to avert nosocomial transmission.

## Limitations

This review is based on the retrospective review of programmatic data of only one humanitarian agency’s cholera responses in three Sub-Saharan African countries. Thus the generalisability of our findings may be limited as we only have limited data from one agency and three countries. The data were neither publicly available nor in a prescribed format for this analysis. The review relied on the manual compilation of reports from the MSF archives in each target country and what was available at the time of request. There is no compilation of cholera responses or cholera data internally in MSF or globally. We therefore expect to have unintentionally excluded some reports in our data harvest from the organisation. The information presented across the reports may reflect biases of the report authors, or areas of emphasis typical of MSF’s organisational culture, to an unquantifiable level. Reports are written subjectively, did not follow a set format nor allow implementers to capture challenges or compare responses against one another. For example, the dates of programme delivery and activities may not be collected systematically, nor the dates recorded accurately. As noted in the search strategy, many of the initial reports retrieved were duplicative as they were from the same outbreaks but written by different authors. This questions if the numerous reports written from one outbreak are an efficient use of human resources, and that there could potentially be other ways of writing to support capturing lessons from outbreak responses [16, 17, 60]. The lack of a systematic structure reduces the utility of the reports to capture useful accounts of responses.

There are many more reports available from DRC than either Malawi or Mozambique. This could potentially skew the results and challenges extracted from these outbreak responses. Whilst DRC has frequent outbreaks and can provide transferable lessons to the sub-Saharan African region, further retrospective reviews of outbreak responses would need to cover a broader geographical range and time frame to draw out generalisable recommendations. Interviews with individuals from operational agencies could also prove useful to further elucidate challenges.

Lastly, whilst our analysis draws out common challenges cited in the reports, it does not take into account the contextual constraints of these countries. The response may have been affected by the under-resourced national public health systems in these three countries, concurrent crises or the epidemiology of the settings, such as previous outbreaks, previous vaccination campaigns or the timing of the epidemics, which could all affect both the epidemic propagation and response efforts.

## Conclusions and recommendations

Documentation and evaluation of cholera responses are limited and heterogenous. The case studies included in this review show that responses to cholera epidemics by MSF have varied in implementation strategy, selection of interventions and have incurred considerable delays from alert to response.

Based on this review, we can make a number of recommendations to improve the development of evidence-based, rapid epidemic cholera control efforts:

- Explore improved models for epidemic preparedness, including rapid mobilisation of supplies and deployment of trained staff;
- Focus on the competencies and processes required to rapidly take decisions on the appropriate package of interventions and modality (e.g., community-based interventions versus CATI delivery);
- Invest in and strengthen partnerships with national and local government and other agencies as part of epidemic preparedness activities and invest in coordination mechanisms;
- Dedicate time and staff to training and supervising health care providers to provide adequate case management to admitted cholera patients;
- Conduct rigorous and structured evaluations of cholera response programmes to understand factors for delays, the interlink between health and WASH interventions and provide evidence-based guidance to programmes;
- Standardise reporting templates within and across countries, and across international humanitarian agencies, to provide consistent and accessible data by internal and external staff and collate learnings.

## Supplementary Information

**Additional file 1.** Preferred Reporting Items for Systematic Reviews and Meta Analyses extension for Scoping Reviews (PRISMA-ScR) checklist.

## Data Availability

The datasets generated and/or analysed during the current study are available from the corresponding author on reasonable request.

## Abbreviations

CHW: Community Health Worker
CTC: Cholera Treatment Centre
CTU: Cholera Treatment Unit
DRC: Democratic Republic of Congo
HCF: Health Care Facility
IPC: Infection, Prevention and Control
MSF: Médecins Sans Frontières
MoH: Ministry of Health
NGO: Non-Governmental Organisation
OCV: Oral Cholera Vaccination
ORP: Oral Rehydration Point
PNECHOL-MD: Programme National d’Elimination du Choléra et de lutte contre les autres Maladies Diarrhéiques
POU: Point of use
RDT: Rapid Diagnostic Test
WASH: Water, sanitation and hygiene
